# Data-Driven Discovery of Predictors of Virtual Reality Safety and Sense of Presence for Children With Autism Spectrum Disorder: A Pilot Study

**DOI:** 10.3389/fpsyt.2020.00669

**Published:** 2020-08-04

**Authors:** Mahan Malihi, Jenny Nguyen, Robyn E. Cardy, Salina Eldon, Cathy Petta, Azadeh Kushki

**Affiliations:** ^1^ Institute of Biomedical Engineering, University of Toronto, Toronto, ON, Canada; ^2^ Autism Research Centre, Bloorview Research Institute, Holland Bloorview Kids Rehabilitation Hospital, Toronto, ON, Canada

**Keywords:** autism spectrum disorder, virtual reality, technology-aided intervention, usability study, sense of presence, oculus, data-driven, machine learning

## Abstract

Virtual reality (VR) offers children with autism spectrum disorder (ASD) an inexpensive and motivating medium to learn and practice skills in a personalized, controlled, and safe setting; however, outcomes of VR interventions can vary widely. In particular, there is a need to understand the predictors of VR experience in children with ASD to inform the design of these interventions. To address this gap, a sample of children with ASD (n=35, mean age: 13.0 ± 2.6 years; 10 female) participated in a pilot study involving an immersive VR experience delivered through a head-mounted display. A data-driven approach was used to discover predictors of VR safety and sense of presence among a range of demographic and phenotypic user characteristics. Our results suggest that IQ may be a key predictor of VR sense of presence and that anxiety may modify the association between IQ and sense of presence. In particular, in low-anxiety participants, IQ was linearly related to experienced spatial presence and engagement, whereas, in high-anxiety participants, this association followed a quadratic form. The results of this pilot study, when replicated in larger samples, will inform the design of future studies on VR interventions for children with ASD.

## Introduction

Autism spectrum disorder (ASD) is a complex neurodevelopmental disorder defined by differences in social communication and the presence of restricted and/or repetitive behaviors (1). ASD is a highly heterogeneous condition with large variability in etiology (2, 3), neurobiology (4, 5), and phenotypic presentation (6). ASD is also associated with several co-occurring conditions such as anxiety, attention-deficit/hyperactivity disorder (ADHD), obsessive-compulsive disorder, epilepsy, and intellectual disability. Variability in the presence and severity of these co-occurring conditions further adds to the heterogeneity of ASD and intervention outcomes.

Timely and appropriate interventions and supports can improve long-term health and societal outcomes for many children with ASD (7, 8); however, most available evidence-based interventions are costly and resource-intensive (e.g., up to 40 h/wk of 1-on-1 therapy). The heterogeneity of ASD necessitates significant personalization of interventions, which further challenge treatment development. Technology-based interventions, if used appropriately, hold significant promise to reduce these barriers. One such technology is virtual reality (VR): computer-generated, interactive environments that simulate the real world by presenting the user with three-dimensional imagery. VR provides an inexpensive way to learn and repeatedly practice skills in a personalized, controlled, and safe setting (9, 10), and can improve ecological validity of interventions and generalizability of learned skills (11, 12). Feasibility studies have demonstrated the potential of VR for skills training across several domains including job interview training (13), vocational training (14), social cognition skills training (15–19), driving simulation (20, 21), and anxiety reduction (22). Despite this promise, the outcomes of VR interventions are highly variable. Moreover, VR use by children may be associated with physical, social, and psychological risks (23) including low therapeutic value, cybersickness, and increased screen time which may lead to social isolation, lack of physical activity, and obesity (24–26). Many VR systems now use head-mounted displays (HMDs), a mode of delivery of VR experiences that relies on glasses-like displays covering the user’s eyes to provide a three-dimensional view of a scene. While HMDs can enhance the sense of presence and immersiveness of VR experiences, their use has been associated with side effects, including cybersickness, a physical condition characterized by eye strain, headache, dizziness, and nausea. There is a significant gap in understanding these risks and predictors of optimal user experiences, especially given the highly diverse needs of children with ASD. This paucity of knowledge is a critical barrier to the implementation of VR for interventions in ASD, and identifying subgroups who may respond to VR in a similar manner will be a necessary precursor to investigations on clinical effectiveness. To address this gap, the present pilot study examined predictors of VR experience (sense of presence and safety) in children with ASD.

VR experience can be quantified through various measures including the sense of presence in the virtual environment (27), the degree of engagement with presented content (28–30), and the perceived ecological validity (31). In the general population, these dimensions of VR experience have been associated with anxiety (32), gender (33), and age (34) among other factors. However, the extent to which these predictors may impact VR experience in children with ASD is not well-understood. It also remains unclear whether additional variables such as ASD symptoms severity, IQ, and attention difficulties may also affect VR responses in children with ASD.

Two factors challenge our study of VR predictors in children with ASD. First, ASD is a multi-faceted condition with differences in several symptom domains that may impact VR experiences. These differences translate into a large number of candidate predictors of VR and limit the statistical power of traditional regression analyses. Second, the large variability across the autism spectrum can result in complex and nonlinear patterns of association between user characteristics and VR experiences. This necessitates the use of analytical tools beyond traditional linear regression methods that allow for the characterization of complex patterns.

To address the above challenges, the present paper proposes a machine-learning-based approach to discovering predictors of VR sense of presence and safety for children with ASD. Machine learning algorithms are powerful tools for discovering patterns of association among sets of variables directly from the data, often without prior assumptions on what these associations may be. When trained on appropriate datasets, these algorithms can capture highly complex and non-linear patterns in high dimensions. These algorithms also allow for effective variable selection, even with modest sample sizes. As such, these approaches hold significant potential as analytical tools for characterizing the heterogeneity in samples of children with ASD (35).

## Materials and Methods

### Participants

The dataset used in this study has been described in our previous work (36). The set contains data from a sample of 35 children and youth (mean age: 13.0 ± 2.6 years; 10 female) with ASD. The inclusion criteria were a clinical diagnosis of ASD, 8–18 years of age, full-scale and verbal IQ greater than 70, and normal or corrected-to-normal hearing and vision. Exclusion criteria were the use of beta-blockers as these affect autonomic responses, and contraindications for use of VR (history of migraines, seizures, vestibular conditions, hypertension, cardiovascular and circulatory diseases, history of difficulty differentiating between reality and fiction, and predisposition to motion sickness).

**Figure 1:**
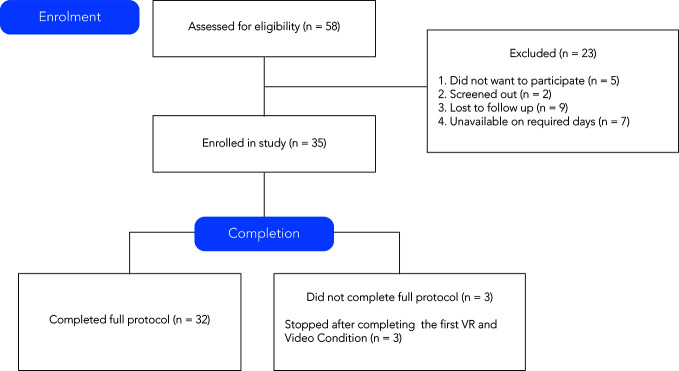
CONSORT (Consolidated Standards of Reporting Trials) (37) chart of recruitment, enrollment, and retention of participants.

Participants were recruited through mail outs and study flyers. **Figure 1** shows the recruitment and screening process. Of the 58 individuals who were interested in the study, 35 were enrolled.

The research ethics board at the Holland Bloorview Kids Rehabilitation Hospital and the University of Toronto approved the study. Participants who were deemed to have the capacity for consent provided written consent. Others provided assent and their legal guardians provided written consent.

### Measures

#### Participant Characteristics

Participants had a clinical diagnosis of ASD, confirmed by the gold-standard instruments: Autism Diagnostic Observation Schedule (ADOS) (38) and the Autism Diagnostic Interview-revised (ADI-R) (39). IQ was measured using the Wechsler Abbreviated Scale of Intelligence (WASI), second edition (40). ASD symptomatology was measured by the Social Communication Questionnaire (SCQ) (41, 42), a 40-item parent-report questionnaire probing ASD-like behaviors. ADHD symptoms were quantified using the attention problems subscale of the Child Behaviour Checklist (CBCL) (43), a parent report questionnaire of behavioral and emotional challenges in children. Baseline anxiety symptom severity was measured using the Screen for Child Anxiety Related Emotional Disorders (SCARED) (44), a 41-item parent report of anxiety symptoms in children.

#### Safety

The VR safety dimensions considered in this study included symptoms of cybersickness and anxiety. Cybersickness symptoms were quantified using the negative effects subscale of the Independent Television Commission—Sense of Presence Inventory (ITC-SoPI) (45), a 39-question self-report that measures four dimensions of user experience on a five-point Likert scale: spatial presence, engagement, ecological validity/naturalness, and negative effects. The ITC-SoPI has substantial psychometric evidence and has been used in previous studies examining the experience of VR in adults (9, 46) and adolescents (47, 48) with ASD. Items on the negative effects subscale probe symptoms related to dizziness, eyestrain, headache, nausea, and tiredness. Anxiety symptoms were quantified using the State-Trait Anxiety Inventory (STAI) (49, 50), which is a 20-question self-report questionnaire. We used the difference between STAI score at baseline and that after VR exposure as our measure of anxiety.

#### Sense of Presence

We quantified users’ sense of presence of the VR experience using three subscales of the ITC-SoPI questionnaire (spatial presence, engagement, and naturalness). The participants were also asked if they prefer the VR condition to the video condition (VR preference; yes/no/neutral).

### Procedures

Participation in this study entailed a single 2–3 h study visit to a research laboratory. The protocol consisted of a VR condition using an HMD (Oculus Rift, 2160 by 1200 resolution) and a monitor-displayed 360° video control condition (ViewSonic VP2468, 1920 by 1080 resolution). The HMD’s integrated audio system and Sony on-ear headphones provided audio for the VR and control conditions, respectively. Both conditions depicted the same 5-min scenario developed by Shaftesbury Films (shaftesbury.ca, 4096 by 2048 resolution, 29.97 frames per second) and in partnership with families and clinicians who had extensive experience with the challenges faced by children with ASD on school buses. The scenario placed the user seated inside a stationary school bus, with a driver and other children on the bus. During the scenario, seven children entered the bus and engaged in verbal interactions among each other. Several sensory and social triggers were presented, including street noise (e.g., sirens, construction equipment), and social stimuli (e.g., children entering the bus, children exhibiting behavioral issues, the driver reprimanding the children).

During the study visit, participants were seated in front of a computer monitor that displayed the stimuli and the study questionnaires (**Supplementary Figure 2**) and familiarized with the study protocol using a visual storyboard. Participants were instructed to explore the scenario by moving their head (VR) or a computer mouse (control). Following an initial baseline task, VR and control conditions were each repeated twice (presentation order of VR or control first was randomized) and separated by a subsequent baseline task in which participants watched 5-min clips from the Blue Planet series on a computer monitor (**Supplementary Figure 1**). The self-report questionnaires were administered throughout the study protocol as follows: the STAI following the initial baseline; STAI following each initial VR and control condition; and the ITC-SoPI following each final VR and control condition (**Supplementary Figure 1**).

### Analyses

Analyses were conducted in Python 3.7.3 using Scikit-learn toolbox (51) and JMP^®^ (Version 13.2.1. SAS Institute Inc., Cary, NC, 1989-2019). The significance level for type I error in all tests was set to α = 0.05.

#### Features

A feature vector with 14 user characteristics that affect VR experiences was associated with each participant (**Supplementary Table 1**). These features were age, sex, IQ (full-scale, verbal, and non-verbal), previous experience with VR (yes or no), SCQ total score, ADHD problems score from CBCL, and SCARED scores (subscales and total score). For the SCARED, subscale scores for panic disorder, generalized anxiety disorder, social phobia, and separation anxiety disorder were binarized based on clinical cut-offs.

#### Top Features

The objective of this study was to identify top predictors of measures of safety (cybersickness, anxiety) and sense of presence (spatial presence, engagement, naturalness, preference) from user characteristics listed in **Supplementary Table 1**. Data-driven regression methods were used to identify predictors of continuous measures (spatial presence, engagement, naturalness) whereas classification was used to determine predictors of user preference (yes/no/neutral). Note that these are different than traditional linear regression analyses. In particular, four data-driven methods were used for both regression and classification. These included regularized linear regression using the elastic net method (52). Regularization can reduce model variability (53) and provides a built-in capacity for variable selection. To this end, we used the magnitude of regression coefficients to compute the importance of each predictor variable in the model (53). Elastic net was used with four combinations of parameters, with α=0 corresponding to traditional linear regression. We also used two ensemble methods namely, AdaBoost (54) and random forests (55) regression. These methods are shown to offer robustness to outliers (56) and enhanced performance compared to other regressors in a wide range of applications (57). They also provide outputs and feature weights that are readily interpretable (52, 55). The importance of each feature in these models was quantified as the permutation accuracy importance (55, 58). Finally, we used a multi-layer perceptron (MLP) as artificial neural networks provide flexibility to capture high complexity in the data (52). When used with a large number of features and layers, these models are difficult to interpret as the estimated parameters of the model, known as weights, are not directly convertible to a meaningful measure of relevance. However, given our simple design, the MLP can be used effectively for feature selection (59). To determine the importance of each feature, the dependent resampled input method was used (60).

Parameters of each model were determined based on a grid search in a leave-one-out cross-validation scheme (61). For the random forest, the criterion was based on the Gini coefficient, and 10 (maximum depth 5) and 25 (maximum depth 5) estimators were used for regression and classification, respectively. For AdaBoost, the weak learners were 20 and 50 decision trees with depths of 3 and 1 for regression and classification, respectively. For the MLP, a two-layer network with three neurons was used. The activation functions were tanh, linear, and Gaussian for regression (one from each) and relu for classification. For each feature, its overall importance on a given model was derived by averaging its importance in that model over all rounds of cross-validation. Significant features were determined using the elbow of the feature importance plot.

#### Evaluation Metrics

The machine learning approaches used here do not directly provide traditional regression statistics (e.g., R^2^, p value for the association of outcomes and predictors). Instead, the performance of these methods is commonly evaluated by examining the statistics for the association between true outcomes and those predicted by the regressor, with the null hypothesis of no association (regression coefficient is equal to zero; prediction are not better than chance). To mitigate the risk of over-fitting, leave-one-out cross-validation was used to evaluate the performance of each model. In this approach, data from all but one user were used to train the model, and the testing was performed on the remaining user data. The process was repeated for each participant, and performance was averaged over all folds of cross-validation.

Classification performance was quantified using precision and balanced accuracy. The latter is the percentage of correctly identified samples, where each sample is weighted according to the inverse prevalence of its true class to take into account class imbalance. To compute an aggregate measure for the three-class problem, the measures were averaged for each pair of classes. To evaluate if classification accuracy was greater than chance, the permutation test was used by training the classifier on 100 sets generated by randomizing the data labels (62).

## Results

### Participants

Three of 35 participants did not complete the full study protocol. Reasons for dropout were seemingly related to the bus scenario (participant had prior negative experiences with school buses, participant was agitated by the whistling sounds in the scenario). As experience measurements of non-completers were incomplete, we were unable to include their data in the sense of presence and safety analyses. Participants’ demographic and phenotypic characteristics are shown below in **Table 1** [see also (36)]. Seventy-four percent of participants preferred the HMD-VR to the video condition.

**Table 1:** Participant Information.

Variable	Median (IQR)
Age (years)	13.5(4.75)
Sex (male: female)	22:10
Verbal IQ	93(18)
Performance IQ	105(23)
Full-scale IQ	103(25)
SCQ	22(8.25)
SCARED	24(16)
Experience with VR (Y/N)	20:12
CBCL—ADHD Problems	66(14)

Values are reported as median (IQR).

### Models

The statistics for the regression line characterizing the association between true values of the sense of presence measures and those predicted by each of the data-driven methods are reported in **Table 2**. For spatial presence, better than chance performance (significant associations between true outcomes and predictions) was achieved by the neural network (R^2^ = 0.4, β=0.42 ± 0.12, p=0.01), random forest (R^2^ = 0.4, β=0.35 ± 0.1, p<0.001), and AdaBoost (R^2^ = 0.4, β=0.37 ± 0.1, p<0.001). For engagement, better than chance performance was achieved by the neural network (R^2^ = 0.5, β=0.39 ± 0.1, p=0.03) and random forest (R^2^ = 0.2, β=0.32 ± 0.1, p=0.02). None of the models were able to predict negative effects or STAI difference.

**Table 2:** Regression results for sense of presence.

	Spatial presence			Engagement			Naturalness		
	R^2^	β ± SD	p	R^2^	β	p	R^2^	β	p
**Elastic net** **(α=0)**	−.05	−.03 ± .1	n.s.	−1.1	−.03 ± .1	n.s.	−1.3	−.03 ± .1	n.s.
**Elastic net** **(α=1, l_1 =_ 0)**	−.1	.09 ± .1	n.s.	−.4	.02 ± .1	n.s.	−.4	−.03 ± .1	n.s.
**Elastic net** **(α=1, l_1 =_ 1)**	−.1	−.02 ± .1	n.s.	−.4	−.03 ± .1	n.s.	−.3	−.03 ± .1	n.s.
**Elastic net** **(α=1, l_1_=.5)**	−.1	−.03 ± .1	n.s.	−.4	−.03 ± .1	n.s.	−.4	−.02 ± .1	n.s.
**Neural network**	**.4**	**.42 ± .1**	**.01**	**.5**	**.36 ± .1**	**.03**	.4	.22 ± .1	n.s.
**Random forest**	**.4**	**.35 ± .1**	**<.001**	**.2**	**.32 ± .1**	**.02**	.1	.26 ± .1	n.s.
**AdaBoost**	**.4**	**.37 ± .1**	**<.001**	**.2**	**.44 ± .1**	**<.01**	.1	.23 ± .1	n.s.

p-values are reported for bolded text under the right-hand "p" column. (n.s., not significant).

For the classification problem, VR preference was predicted with better than chance accuracy by the random forest (permutation test; p=0.03). None of the other classifiers provided greater than chance accuracy (**Supplementary Table 2**; p=0.03).

### Top Features

For each regressor, the importance of user characteristics was computed as described in *Analyses*. The elbow in the scores plot occurred after the top two or three features for each regressor (**Supplementary Figure 3**). IQ consistently ranked as a top predictor of the sense of presence outcomes in all models except in one, followed by the SCARED scores.

**Figure 2:**
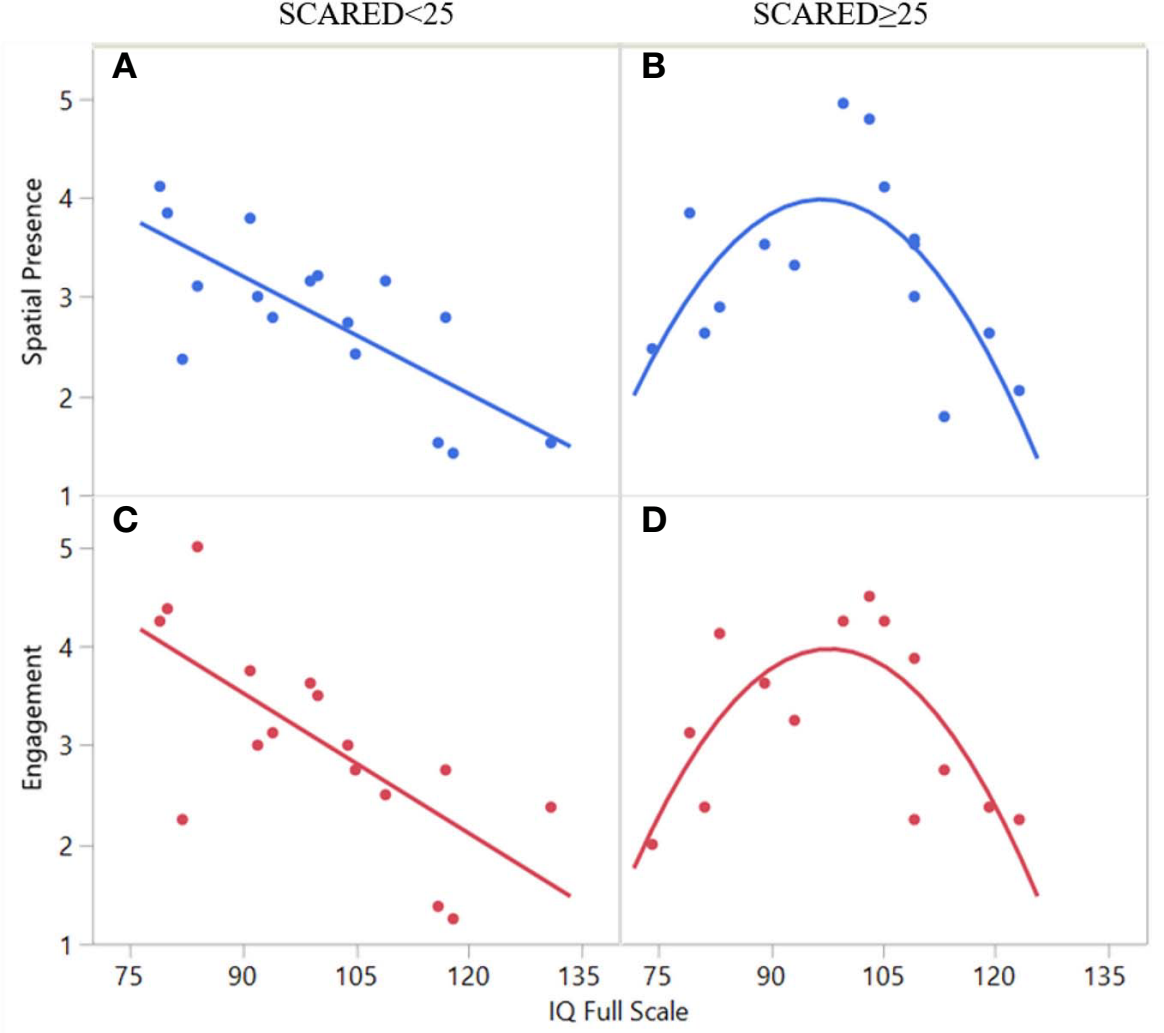
The association between full-scale IQ and spatial presence/engagement in two subgroups of participants: panels **A** and **C** depict a linear association for participants without anxiety (SCARED>25) and panels **B** and **D** depict a quadratic function for participants with anxiety (SCARED>25).

**Figure 2** revealed two subgroups stratified by SCARED score: the first group, characterized by below-threshold score on the SCARED (n=16; SCARED<25), showed a linear association between IQ and spatial presence/engagement (spatial presence: R^2^ = 0.56, β=−0.07 ± 0.01, p=0.008; engagement: R^2^ = 0.50, β=−0.05 ± 0.01, p=0.002). For the second subgroup (n=15; SCARED≥25), the association between IQ and spatial presence/engagement, followed a quadratic function (spatial presence: R^2^ = 0.49, β_1_=−0.019 ± 0.012, β_2_=−0.003 ± 0.000, p=0.02; engagement: R^2 =^ 0.55, β_1_=−0.012 ± 0.011, β_2_=−0.003 ± 0.000, p=0.007). Detailed statistical results are presented in **Supplementary Table 3**.

## Discussion

In this pilot study, we used data-driven approaches to discover predictors of VR safety and sense of presence for children with ASD. These approaches have the potential to address the complexity that arises from the large variability in the characteristics of children with ASD. They are also particularly useful for generating hypotheses regarding key predictors among a large number of demographic and phenotypic characteristics that may impact the VR experience. In particular, we examined four models for predicting user-reported ratings of sense of presence (spatial presence, naturalness, engagement) and safety (cybersickness, anxiety). These included elastic nets, random forests, AdaBoost, and neural networks. The neural network, random forest, and AdaBoost performed provided the best predictions of experience outcomes, suggesting a non-linear association between predictors and sense of presence outcomes.

The most accurately predicted target across all models was spatial presence, followed by engagement. Both of these variables are important dimensions of VR experiences that have been associated with response to VR interventions (63). Sense of presence is a “psychological state in which virtual objects are experienced as actual objects in either sensory or nonsensory ways” (64). This key aspect of VR experience has shown to predict responses to VR interventions by facilitating knowledge acquisition and transferability to real environments (65). Engagement reflects attention to the virtual stimuli and has been associated with enhanced intervention effectiveness (66). IQ (full-scale, verbal, performance) and anxiety traits (measured by the SCARED) were identified as key predictors of spatial presence and engagement across different estimators. One estimator (random forest) also identified attention (as measured by the CBCL) as a predictor of spatial presence. Only one other study has considered the role of IQ in VR, suggesting that IQ is not a predictor of willingness to use HMDs (46). The results of our study highlight the role of IQ as an important consideration in VR use. Future studies are needed to replicate and further examine this issue.

The association of anxiety with spatial presence was not surprising as previous studies in other populations have reported an increased sense of presence with heightened state anxiety (67). One explanation for this association may be increased vigilance during states of anxiety. Interestingly, our data-driven approach suggested that anxiety may modify the association between IQ and sense of presence. In particular, we identified two types of associations between spatial presence/engagement and IQ, depending on whether or not a participant had clinically significant anxiety based on the SCARED. For the group of participants without anxiety, spatial presence/engagement were negatively associated with IQ in a linear fashion. This negative linear association may be related to the enhanced ability of participants with higher IQ scores to differentiate between the real and virtual worlds. Our results suggest that anxiety may change the association between presence/engagement and IQ from a linear to a quadratic function, suggesting that the presence of anxiety in participants with lower IQs may decrease the sense of presence. In the high-anxiety group, those with IQ scores in the average range (85–115) reported the highest experience of spatial presence/engagement.

None of the methods used in this study were able to successfully predict cybersickness or anxiety experienced during the VR emersion. User experiences of VR are thought to be impacted by both user characteristics as well as factors extrinsic to the user such as system characteristics, task characteristics, and media content (67). As this study only considered the user characteristics, it may be possible that these negative effects can be better predicted by external factors such the degree and type of motion in the VR experience or the duration of exposure (68, 69). Future studies are needed to further investigate the role of these factors.

Another unexpected finding in this study was that, in contrast to other studies of the general population, our methods did not identify age and sex as significant predictors of cybersickness. This may be related to our narrow age range (70–72) or sex differences in ASD.

The findings of this pilot study must be interpreted in the context of several limitations. First, our modest sample size may have resulted in overfitting of our complex machine learning models. Second, given our recruitment strategy, individuals with previous negative VR experiences may not have self-identified to partake in the study. This may have resulted in sampling bias and overly optimistic outcomes. Third, we were unable to obtain outcome measures for participants who did not complete the study and our conclusions are limited to the completer sample. Future studies with an intent-to-treat design are needed to further examine the safety and tolerability in non-completers. Fourth, our study only included participants without intellectual disability. Future studies are needed to examine VR safety and tolerability in a more diverse sample. Fifth, this study only considered a limited set of user characteristics. Future studies are needed to examine the impact of other characteristics, such as sensory differences, on VR experiences. Lastly, the VR content tested in the study was limited to only one, short scenario. Long-term exposure to different types of VR content (e.g., high motion) may be associated with other predictors of safety and sense of presence. Consequently, the interaction of user characteristics and VR content features was not considered; future studies with multiple scenarios may be able to elucidate these effects. In the context of long-term exposure, other measures of safety, such as dependency, decrease in physical activity, and sleep difficulties should be considered.

### Implications

To our knowledge, this pilot, hypothesis-generating study is the first to examine predictors of VR safety and sense of presence in children with ASD. Through a data-driven approach, we identified IQ and anxiety to important variables for consideration in future studies of VR usability in this population.

## Conclusion

We examined the effect of user characteristics on sense of presence and safety of VR for children with ASD. Given the heterogeneity in ASD, we employed a data-driven approach based on machine learning. Our results suggest that IQ and anxiety may affect VR usability in this sample.

## Data Availability Statement

The datasets generated for this study will not be made publicly to available because public disclosure of data was not included in the ethics approval and participant informed consent.

## Ethics Statement

This study involving human participants was reviewed and approved by Holland Bloorview Kids Rehabilitation Hospital. Written informed consent to participate in this study was provided by participants who were deemed to have the capacity for consent, others provided assent and their legal guardians provided written informed consent.

## Author Contributions

MM led the data analysis, interpretation of results, and drafted the manuscript. JN contributed to conceptualization of the protocol, data collection, data analysis, and drafting of the manuscript. RC contributed to the interpretation of results and drafting of the manuscript. SE and CP conceptualized and designed the study and contributed to interpretation of the results and drafting of the manuscript. AK conceptualized and designed the study, and contributed to data analyses, interpretation of the results, and drafting of the manuscript.

## Funding

The study was funded by the Natural Sciences and Engineering Research Council of Canada (NSERC) and Ontario Centres of Excellence (OCE) grant number 26308. Shaftesbury, a media company developing VR experiences for children, including those with ASD, contributed to NSERC/OCE funding. MM was funded by the Holland Bloorview Foundation Graduate Student Scholarship.

## Conflict of Interest

This study was partially funded by Shaftesbury, who may benefit financially from commercialization of virtual reality experiences for children with ASD. AK serves on the Board of Advisors for Shaftesbury Technology.

The remaining authors declare that the research was conducted in the absence of any commercial or financial relationships that could be construed as a potential conflict of interest.
