# Supplementary material for: Data-Driven Discovery of Predictors of Virtual Reality Safety and Sense of Presence for Children With Autism Spectrum Disorder: A Pilot Study
Source: Front Psychiatry. 2020 Aug 4;11:669. doi: 10.3389/fpsyt.2020.00669 (PMC7438752; doi:10.3389/fpsyt.2020.00669)
Supplement: Supplementary file 1 [file DataSheet_1.docx]

# Supplementary Information

**Supplementary Table 1. Description of predictors.**

| Predictors | Description | Variable type |
| --- | --- | --- |
| Age | Age in years | Integer (range: 8 - 17) |
| Gender | Participant gender | Binary (male/female) |
| Anxiety traits (SCARED) | Anxiety disorder (total score) | Integer (range: 8-42; clinical cut-offs: 25 and 30) |
|  | Panic disorder (or significant somatic symptoms) | Integer (range: 0-8; clinical cut-off: 7) |
|  | Generalized anxiety disorder | Integer (range: 0-16; clinical cut-off: 9) |
|  | Separation anxiety disorder | Integer (range: 0-10; clinical cut-off: 5) |
|  | Social anxiety disorder | Integer (range: 0-14; clinical cut-off: 8) |
|  | School avoidance | Integer (range: 0-7; clinical cut-off: 3) |
| Attention (CBCL) | ADHD problems | Integer (range: 50-78; clinical cut-off: 65) |
| ASD Symptoms (SCQ) | Total score | Integer (range: 6-29; clinical cut-off: 15) |
| IQ (WASI) | Full-scale | Integer (range: 74-131) |
|  | Verbal | Integer (range: 74-127) |
|  | Non-verbal | Integer (range: 64-138) |
| Previous VR experience | Participant’s previous experience with VR | Binary (yes/no) |

Supplementary Table 2. Classification results for preference question in different models using all/top features.

|  | I preferred a VR bus to watching the video of a school bus | | | |
| --- | --- | --- | --- | --- |
|  | All features | | Top features | |
|  | Precision | Balanced Accuracy | Precision | Balanced Accuracy |
| Elastic net  (α=0) | 0.5 | 0.3 | - | - |
| Elastic net  (α=1, l_1_=0) | 0.3 | 0.2 | - | - |
| Elastic net  (α=1, l_1_=1) | 0.4 | 0.3 | - | - |
| Elastic net  (α=1, l_1_=.5) | 0.3 | 0.3 | - | - |
| Neural network | 0.7 | 0.4 | 0.8 | 0.5 |
| Random forest | 0.8 | 0.5 | **0.8** | **0.8** |
| AdaBoost | 0.7 | 0.5 | 0.7 | 0.4 |

Supplementary Table 3. Results of linear and quadratic models for predicting spatial presence and engagement by full-scale IQ.

| Target | Model | SCARED score <25 (N=16) | | | SCARED score ≥25 (N=15) | | |
| --- | --- | --- | --- | --- | --- | --- | --- |
|  |  | R^2^ | RMSE | P-value | R^2^ | RMSE | P-value |
| Spatial Presence | Linear | 0.56 | 0.55 | 0.008 | 0.015 | 0.94 | 0.6534 |
|  | Quadratic | 0.57 | 0.57 | 0.0038 | 0.49 | 0.70 | 0.0170 |
| Engagement | Linear | 0.50 | 0.75 | 0.0021 | 0.00 | 0.90 | 0.9579 |
|  | Quadratic | 0.51 | 0.77 | 0.0096 | 0.55 | 0.62 | 0.0074 |


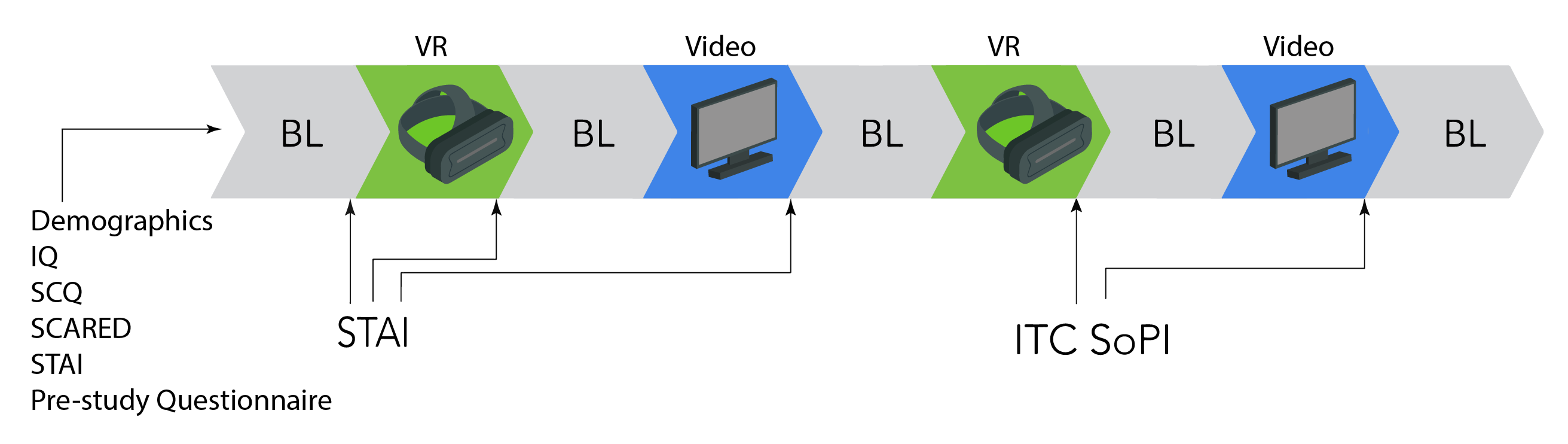


Figure 1. Timing and order of questionnaire administration (BL: baseline, VR: virtual reality, SCQ: Social Communication Questionnaire, SCARED: Screen for Anxiety Related Emotional Disorders; STAI: State-Trait Anxiety Inventory, ITC-SoPI: Independent Television Commission – Sense of Presence Inventory).


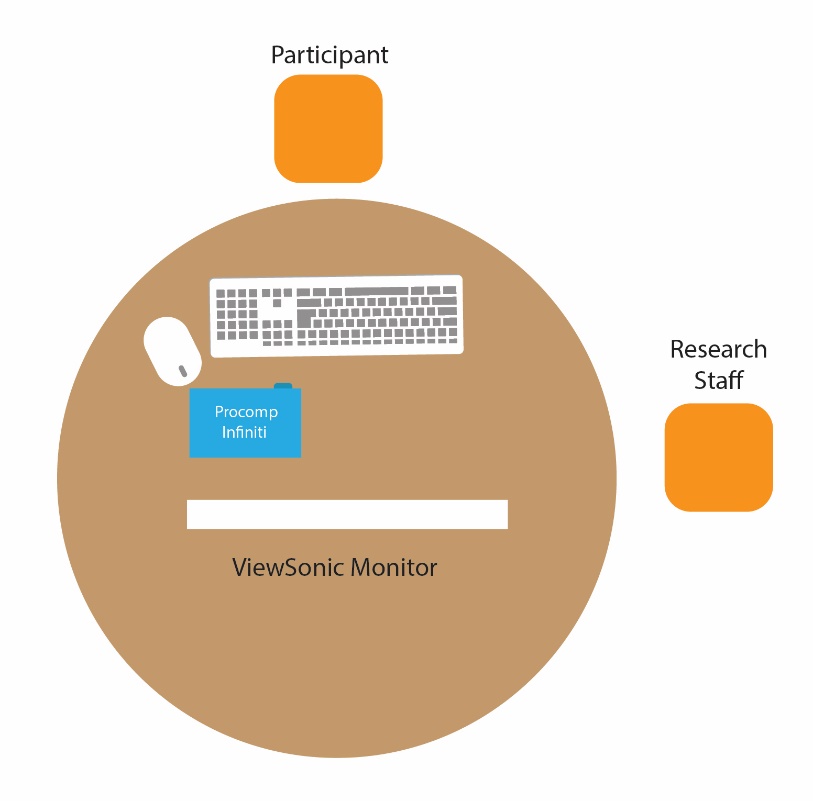


Figure 2. Schematic of the experiment room.


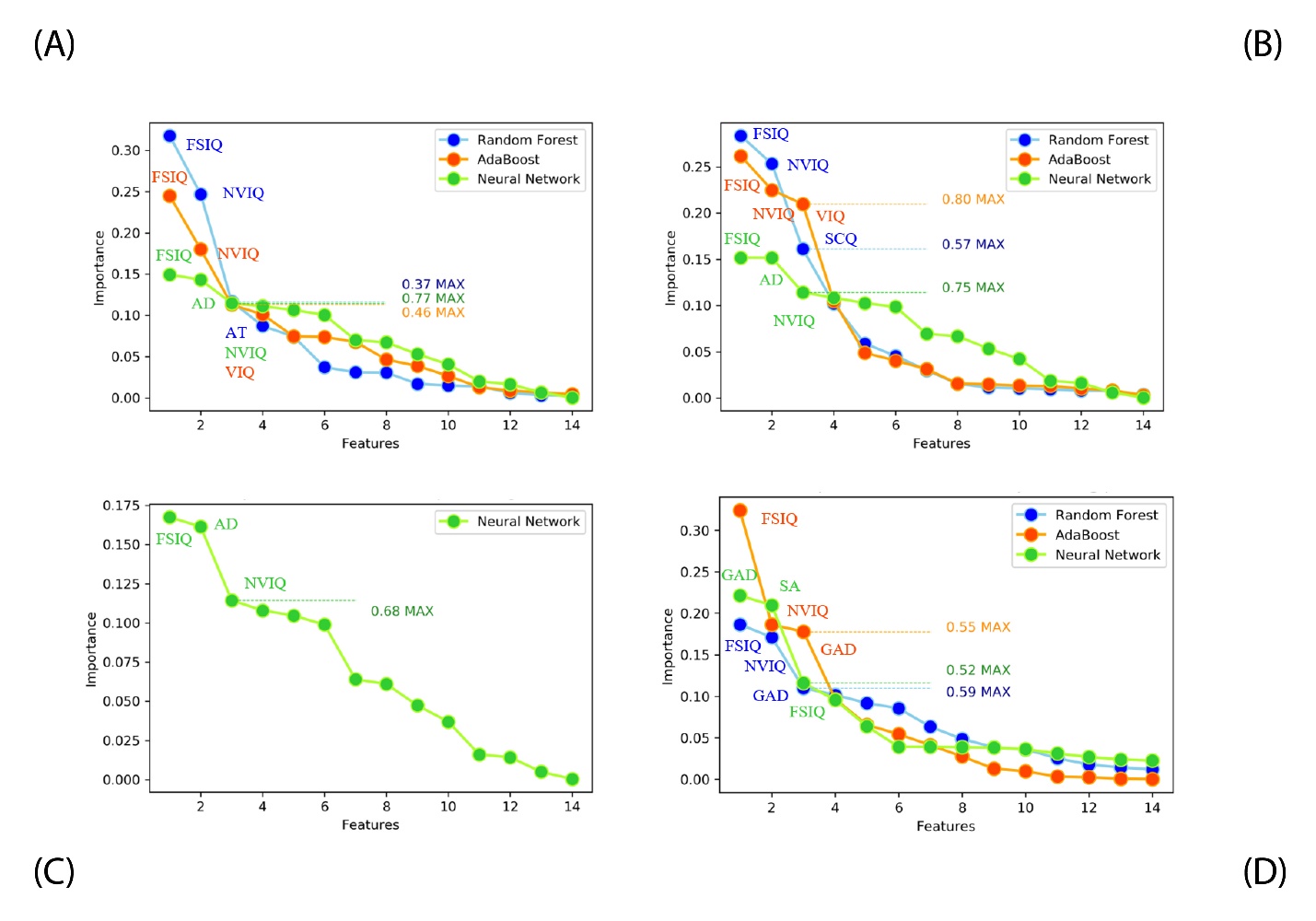


Figure 3. Ordered feature importance: (A) spatial presence, (B) engagement, (C) naturalness, (D) preference. MAX indicated the ratio between the importance scores for the 1st and 3rd features. FSIQ: Full-scale IQ, VIQ: Verbal IQ, NVIQ: Non-verbal IQ, AD: Anxiety Disorder, AT: Attention, GAD: Generalized Anxiety Disorder, SA: School Avoidance.
